# Antivirulence effects of cell-free culture supernatant of endophytic bacteria against grapevine crown gall agent, *Agrobacterium tumefaciens*, and induction of defense responses in plantlets via intact bacterial cells

**DOI:** 10.1186/s12870-024-04779-1

**Published:** 2024-02-10

**Authors:** Faegheh Etminani, Behrouz Harighi, Bahman Bahramnejad, Ali Akbar Mozafari

**Affiliations:** 1 Department of Plant Protection, Faculty of Agriculture, University of Kurdistan, Sanandaj, Iran; 2 Department of Plant Production and Genetics, Faculty of Agriculture, University of Kurdistan, Sanandaj, Iran; 3 Department of Horticultural Science, Faculty of Agriculture, University of Kurdistan, Sanandaj, Iran

**Keywords:** *Agrobacterium tumefaciens*, Biological control, Endophytic bacteria, Crown gall, Non-volatile compounds, Virulence traits

## Abstract

**Background:**

Crown gall disease caused by *Agrobacterium tumefaciens* is a very destructive affliction that affects grapevines. Endophytic bacteria have been discovered to control plant diseases via the use of several mechanisms. This research examined the potential for controlling crown gall by three endophytic bacteria that were previously isolated from healthy cultivated and wild grapevines including *Pseudomonas kilonensis* Ba35, *Pseudomonas chlororaphis* Ba47, and *Serratia liquefaciens* Ou55.

**Result:**

At various degrees, three endophytic bacteria suppressed the populations of *A. tumefaciens* Gh1 and greatly decreased the symptoms of crown gall. Furthermore, biofilm production and motility behaviors of *A. tumefaciens* Gh1were greatly inhibited by the Cell-free Culture Supernatant (CFCS) of endophytic bacteria. According to our findings, CFCS may reduce the adhesion of *A. tumefaciens* Gh1 cells to grapevine cv. Rashe root tissues as well as their chemotaxis motility toward the extract of the roots. When compared to the untreated control, statistical analysis showed that CFCS significantly reduced the swimming, twitching, and swarming motility of *A. tumefaciens* Gh1. The findings demonstrated that the endophytic bacteria effectively stimulated the production of plant defensive enzymes including superoxide dismutase (SOD), polyphenol oxidase (PPO), peroxidase (POD), phenylalanine ammonia lyase (PAL), and total soluble phenols at different time intervals in grapevine inoculated with *A. tumefaciens* Gh1. The Ba47 strain markedly increased the expression levels of defense genes associated with plant resistance. The up-regulation of *PR1*, *PR2*, *VvACO1*, and *GAD1* genes in grapevine leaves indicates the activation of SA and JA pathways, which play a role in enhancing resistance to pathogen invasion. The results showed that treating grapevine with Ba47 increased antioxidant defense activities and defense-related gene expression, which reduced oxidative damage caused by *A. tumefaciens* and decreased the incidence of crown gall disease.

**Conclusion:**

This is the first study on how *A. tumefaciens*, the grapevine crown gall agent, is affected by CFCS generated by endophytic bacteria in terms of growth and virulence features. To create safer plant disease management techniques, knowledge of the biocontrol processes mediated by CFCS during microbial interactions is crucial.

**Supplementary Information:**

The online version contains supplementary material available at 10.1186/s12870-024-04779-1.

## Background

One of the most significant plant bacterial diseases in the world is crown gall disease. There have been reports of impairment of nutrient uptake, plant growth, and production in the early stages of infection and severe economic losses in the latter stages of infection up to total plant death [1]. By transferring (T)-DNA from bacterial cells into the genome of the host plant, *A. tumefaciens* induces crown gall disease. The complicated process of *Agrobacterium*-plant interaction involves modifications of the host plant's metabolism and gene expression patterns. Crown galls proliferate and develop more readily when phytohormone levels are higher [2]. Early stages of infection and the relationship between bacteria and plant hosts have been linked to motility, chemotaxis, biofilm formation, and eventual attachment [3].

Crown gall disease is one of the most difficult diseases to treat with chemicals and physiological techniques. Employing resistant plant cultivars and antagonistic microbial species seems to be an effective method to reduce crown gall disease because different defensive response pathways are engaged in the early stages of infection, depending on the plant resistance [4]. Numerous laboratories have reportedly tried to biologically control crown gall disease [5–7]. According to a previous publication, *Agrobacterium vitis* E26, a nonpathogenic strain, has the capacity to produce Ar26, an antibacterial compound that significantly inhibits the growth of *A. vitis* MI3-2 and *A. tumefaciens* CY4 on culture media [8]. The release of antimicrobial active chemicals, competition for nutrition and space, hyper-parasitism, and activation of systemic resistance responses in the host plant are some of the aspects that primarily influence the antagonistic action against bacterial pathogens [9].

In addition to serving a variety of activities including promoting plant development, acting as a biocontrol agent, and adjusting the plant's systemic resistance, endophytic bacteria may live in plants without harming the host. Secondary metabolites produced by endophytic bacteria have the ability to activate plant defense enzymes, which in turn may induce systemic resistance [10]. A positive correlation has been seen between increased host tolerance to pathogenic stress and the activity of vital plant defense enzymes [11]. Endophytic bacteria are a good option to activate systemic resistance to plant pathogens when they colonize the interior tissue of the plant [12]. The production of phenols, the build-up of peroxidase (PO), polyphenol oxidase (PPO), phenyl ammonia lyase (PAL), and superoxide dismutase (SOD), together with the expression of a number of defense-related genes, are all linked to induced systemic resistance (ISR) [13]. Numerous investigations have shown the function of pathogenesis-related (PR) proteins, oxidative enzymes and their metabolic products, and phenolic compound buildup in the defensive mechanisms of diseased plants [14].

In response to pathogen infections, plants often produce a broad variety of PR proteins. Numerous species of mono- and dicotyledonous plants have been reported to have PR proteins [15]. Members of PR1 were shown to have inhibitory action against bacterial pathogens, and a variety of unidentified biological roles [16]. Through the modification of plant immune systems, ethylene plays a crucial part in controlling the colonization of plants by bacteria. Multi-gene families encode 1-Aminocyclopropane-1-Carboxylic Acid Oxidase (ACO), one of the essential enzymes of ethylene production in higher plants [17]. Certain members of the bacterial population associated with plants have the capacity to regulate the amounts of ethylene and ACO in plants, which in turn may alter the defense way in which plants respond to biotic stress [18]. At least three *VvACO* genes have been found in grapes [19]. One important enzyme that catabolized glutamate to gamma-aminobutyric acid (GABA) is glutamate decarboxylase (GAD). In particular, glutamate decarboxylase (GAD) is critical for resistance to stress [20]. Plant-derived GABA stimulates quorum quenching in *Agrobacterium* during plant-bacterium interaction, which reduces bacterial pathogenicity [21].

In earlier research, we revealed that some endophytic bacterial strains previously isolated from domesticated and wild grapevine plants inhibited *A. tumefaciens* growth in vitro [22]. The current investigation assessed the impact of CFCS produced by endophytic bacteria on the growth rate of *A. tumefaciens*, structural alteration, and virulence characteristics, including motility, chemotaxis, attachment, and biofilm formation. Additionally, in grapevine (cv. Rashe), the effects of endophytic bacterial suspensions on defense-related enzymes and non-enzyme substances, as well as alterations in the gene expression of two pathogenesis-related genes (*PR1* and *PR2*), *VvACO1*, and *GAD1* genes were examined.

## Results

### Molecular identification of endophytic bacteria

**Fig. 1:**
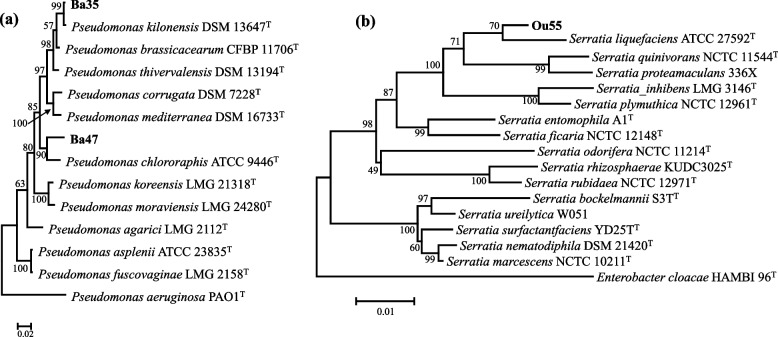
Phylogenetic tree of partial *16S rRNA* and *rpoD* gene sequences indicating the position of endophytic bacteria belonging to the *Pseudomonas* genus (**a**), as well *16S rRNA* and *pgi* gene sequences analysis of strain belonging to *Serratia* genus (**b**) (shown in bold) in addition to taxonomically similar selected reference strains. The analysis was conducted by the Maximum Likelihood method with Tamura-Nei calculation model in MEGA version 6.0. The scale bar shows the number of substitutions per site. Numbers at branching points indicate bootstrap value derived from 1000 replicates

The acquired nucleotide sequences for the *rpoD* and *pgi* genes were deposited in the NCBI nucleotide sequence database with accession numbers OQ657168–OQ657169 and OQ657170, respectively. A phylogenetic analysis of near-complete *16S rRNA* and *rpoD* gene sequences, together with nucleotide identity, revealed that the isolates Ba35 and Ba47 belonged to the *Pseudomonas* genus and had strong similarities with *P. kilonensis* and *P. chlororaphis*, respectively (Fig. 1a). Isolate Ou55 belonged to the *Serratia* genus and had a high degree of similarity with *S. liquefaciens*, according to analysis of the *16S rRNA* and *pgi* gene sequences (Fig. 1b).

### In vitro antibacterial activity of CFCS

**Fig. 2:**
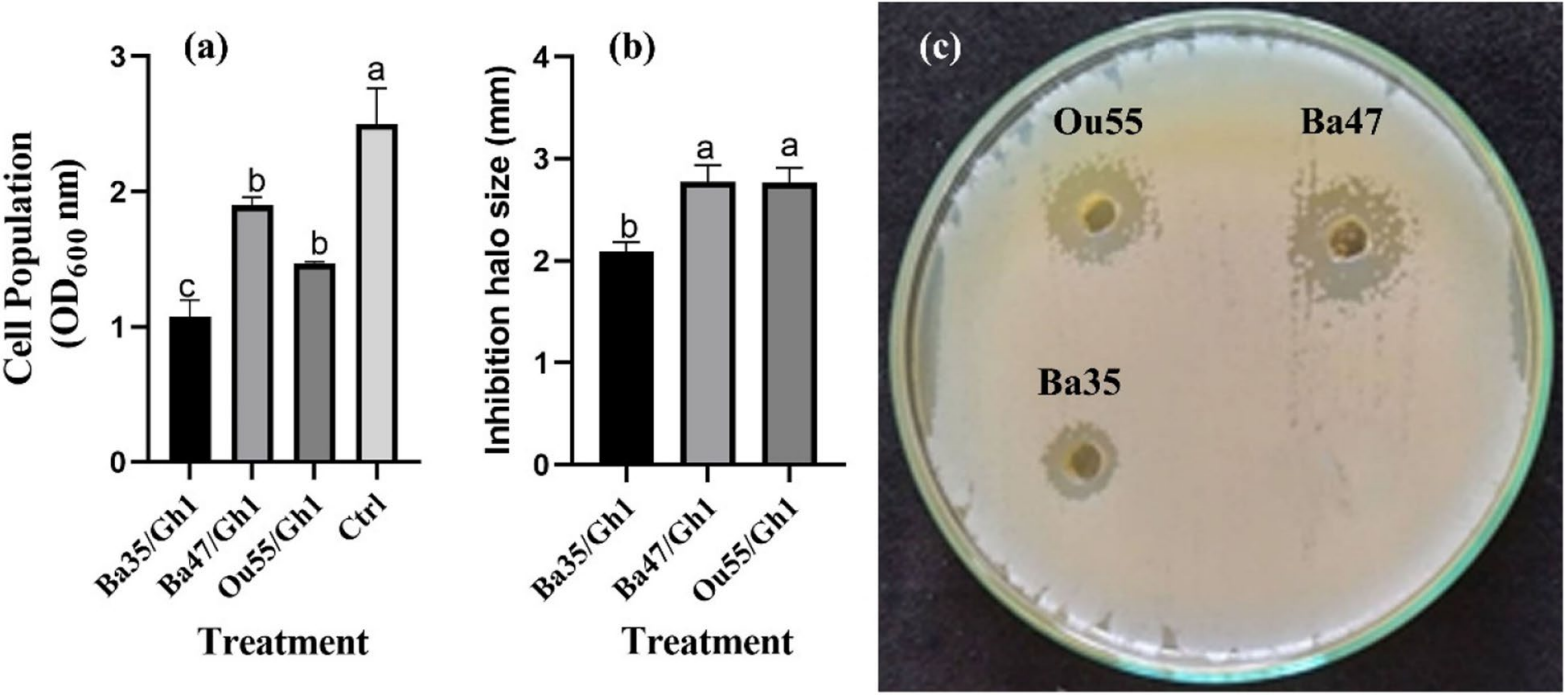
In vitro antagonistic activity of CFCS produced by *Pseudomonas kilonensis* Ba35, *Pseudomonas chlororaphis* Ba47, and *Serratia liquefaciens* Ou55 against *A. tumefaciens* Gh1 compared to the non-treated control (Ctrl). The reduction of cell population (**a**), the inhibition halo size (**b**), and representative plate assay (**c**) were shown. Three replicates were used for each treatment. Error bars indicate SE of the three replicate. Different letters indicate significant differences (*P* = 0.05)

Significant differences were seen in the cell population between all treatments and the non-treated control (F = 16.69, *P* < 0.0001), according to the findings of the ANOVA analysis (Table 1). The Ba35 and Ou55 strains produced CFCS that reduced the *A. tumefaciens* Gh1 cell population by about 57.08% and 41.2%, respectively, with Ba47 producing the lowest decrease at 24% (Fig. 2a). Similarly, Ba35, Ba47, and Ou55's CFCS demonstrated potent antagonistic action against *A. tumefaciens* Gh1 in vitro (Table 1). The Ba47, Ou55, and Ba35 strains had mean inhibitory zone diameters of 2.77, 2.76, and 2.09 mm, respectively (Fig. 2b & c).

**Table 1:** Analysis of variance (ANOVA) of growth inhibition, cell population, biofilm production, swarming-, swimming-, and twitching- motility of *Agrobacterium tumefaciens* Gh1 treated by CFCS of endophytic bacteria

Source of variation	df	inhibition zone (mm)	Cell population (OD_600_ nm)	Biofilm	Swarming	Swimming	Twitching	Chemotaxis (10^6^ cfu/ml)
Treatment	3	0.45*	1.120**	0.54**	31.48**	30.65**	34.96**	146.87**
Erorr	8	0.057	0.067	0.011	0.43	2.16	0.72	6.22
Cv (%)		9.41	14.92	11.87	6.80	13.54	8.60	10.75
F-value		7.98	16.69	49.27	72.54	14.14	48.54	23.61

*df* Degrees of Freedom, *Cv* Coefficient of variation

^*,**^ Significant at 1% and 5% probability level, respectively

### Mode of action of CFCS against *A. tumefaciens* Gh1

#### Effect on motility

**Fig. 3:**
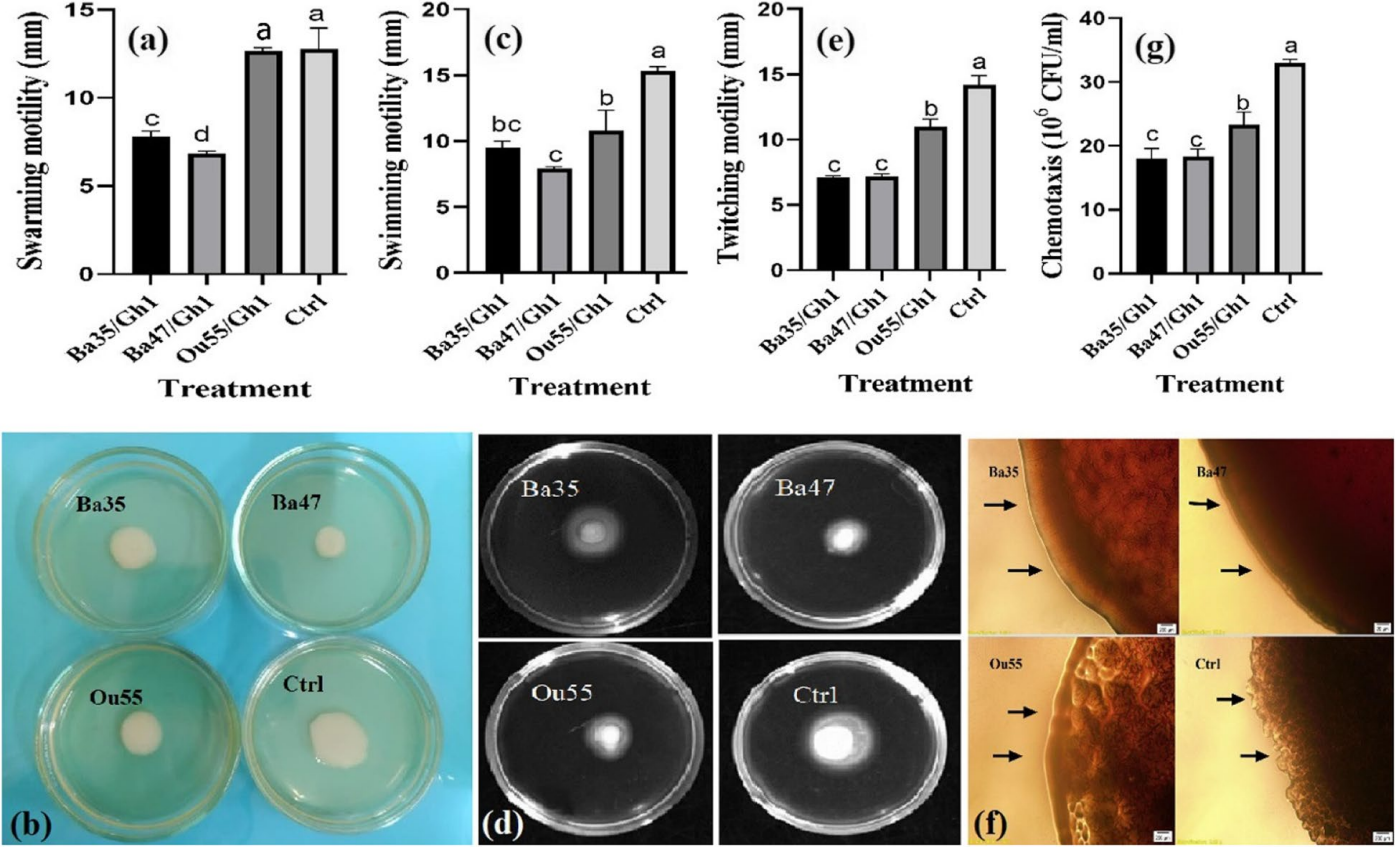
Effect of CFCS produced by *Pseudomonas kilonensis* Ba35 and *Pseudomonas chlororaphis* Ba47, and *Serratia liquefaciens* Ou55, on swarming, swimming, twitching, and chemotaxis motility of *A. tumefaciens* Gh1, compared to the non-treated control (Ctrl). The diameter of swarming motility zone (**a**), and representative plate of swarming motility assay (**b**), the diameter of swimming motility zone (**c**), and representative plate of swimming motility assay (**d**), the diameter of the twitching motility zone (**e**), and representative microscopic examination of the peripheral edge of twitching motility (**f**), and the number of cells attracted toward root extract of grapevine cv. Rashe (**g**) were shown. Three replicates were used for each treatment. Error bars indicate the SE of the three replicates. Different letters indicate significant differences (*P* = 0.05)

The swarming (F = 72.54; *P* < 0.0001), swimming (F = 14.14; *P* < 0.0001), and twitching (F = 48.54; *P* < 0.0001) motility tests showed statistically significant changes between treatments (Table 1). The swarming motility of *A. tumefaciens* Gh1 was considerably decreased by the CFCS generated by all strains of endophytic bacteria, with the exception of Ou55. As shown in Fig. 3a & b, as compared to the control (14.12 mm), Ba47 with a mean of 6.83 mm exhibited the most inhibitory impact, followed by Ba35 (7.79 mm).

The swimming motility of *A. tumefaciens* Gh1 was significantly reduced by the CFCS. In comparison to the control (15.33 mm), Ba47 with a mean of 7.91 mm had greater effects than Ba35, Ou55, and Ba47 with means of 9.47 and 10.78 mm, respectively, as shown in Fig. 3c & d.

Furthermore, the twitching motility of *A. tumefaciens* Gh1 was markedly decreased by the CFCS generated by endophytic bacteria. In comparison to the control (14.18 mm), Ba35 and Ba47, with 7.08 and 7.16 mm, respectively, displayed higher decrease effects, followed by Ou55 with a mean of 11 mm (Fig. 3e). The circumferential colony edge of *A. tumefaciens* Gh1 in the non-treated control was significantly wider, irregular, and lobate, according to microscopic analysis of the twitching motility colonies. In contrast, the entire-smooth colony edge of *A. tumefaciens* Gh1 was observed to be more uniform in cells treated with the CFCS of Ba35, Ba47, and Ou55 (Fig. 3f).

ANOVA analysis results (Table 1) demonstrated that *A. tumefaciens* Gh1 cells treated with CFCS exhibited significantly reduced chemotaxis motility, as measured by the number of cells migrating toward the grapevine (cv. Rashe) root extract, in comparison to the non-treated control (F = 23.61, *P* < 0.0001). Moreover, in comparison to the control, our results indicated that the CFCS of strains Ou55, Ba47 with 45.33% and 44.52%, respectively, had greater reducing effects followed by Ba35 with 29.38% (Fig. 3g).

#### Effect on biofilm formation and grapevine root attachment

**Fig. 4:**
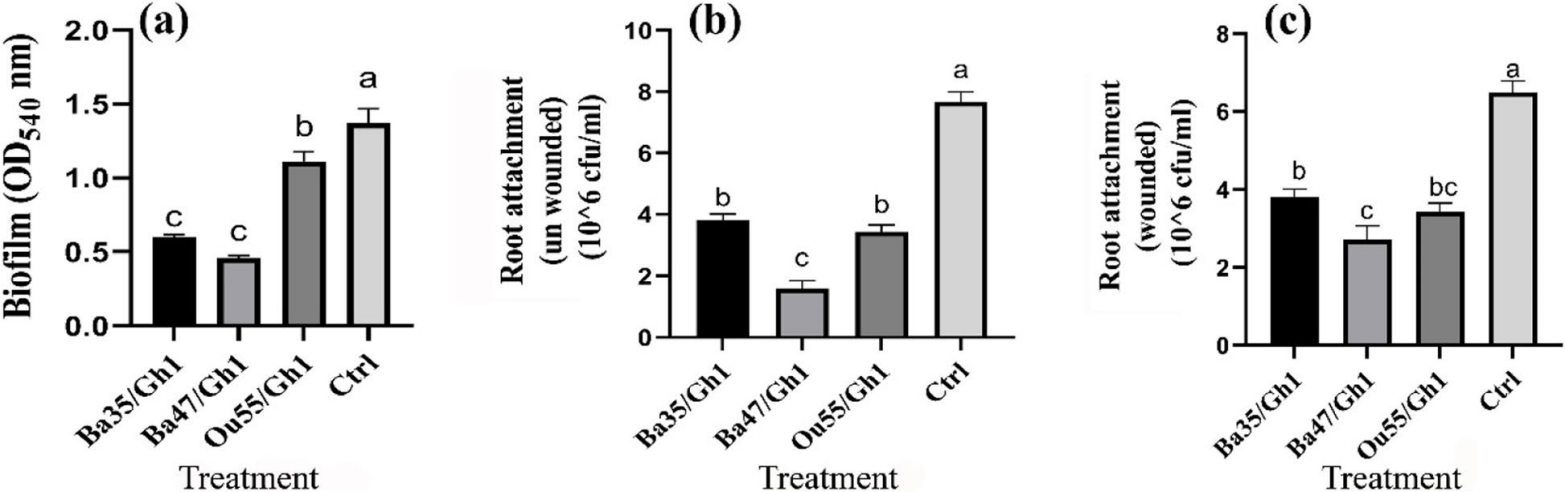
Effect of CFCS produced by *Pseudomonas kilonensis* Ba35, *Pseudomonas chlororaphis* Ba47, and *Serratia liquefaciens* Ou55, on (**a**) biofilm formation of *A. tumefaciens* Gh1, attachment of cells to (**b**) un-wounded root, and (**c**) the wounded root of grapevine cv. Rashe, compared with non-treated control (Ctrl). The graph represents the mean of three replicates. Error bars indicate the SE of three replicates. Different letters indicate significant differences (*P* = 0.05)

In the biofilm formation experiment, there were significant differences between all treatments and the non-treated control (F = 49.27, *P* < 0.0001), according to the findings of the ANOVA analysis (Table 1). Figure 4a demonstrate that CFCS of Ba47 and Ba35 strains had reduction effects of around 66.42% and 56.20%, respectively, whereas Ou55 produced CFCS with a decrease of 18.98%.

Based on the findings of ANOVA (Table 2) there were significant differences between all treatments in the way that *A. tumefaciens* Gh1 cells attached to the wounded (F = 36.45, *P* < 0.0001) and unwounded (F = 96.43, *P* < 0.0001) roots of grapevine cv. Rashe. In comparison with non-treated control, in the unwounded root experiment the highest decrease was associated with Ba47 (79.11%), followed by Ou55 (55.22%) and Ba35 (50.39%) reduction effects (Fig. 4b). Furthermore, in the wounded root, the largest decrease was associated with Ba47, with about 58.46% reduction, followed by Ou55 and Ba35, with 47.23% and 41.53% reduction, respectively (Fig. 4c).

**Table 2:** Analysis of variance (ANOVA) of the effect of CFCS produced by endophytic bacteria on root attachment, gall weight, and biomass production by *Agrobacterium tumefaciens* Gh1

Source of variation	df	wounded root attachment (10^6^ cfu/ml)	unwounded root attachment (10^6^ cfu/ml)	Gall weight (g)	Root fresh weight (g)	Root Dry weight (g)	Shoot fresh weight (g)	Shoot Dry weight (g)	Root length (cm)	Shoot length (cm)
Treatment	3	8.25**	19.49**	0.0013**	62.14	16.19**	51.56**	18.31**	71.93**	475.56**
Erorr	8	0.22	0.202	0.00001	3.55	0.65	5.15	1.17	7.84	54.06
Cv (%)		11.57	10.89	11.11	11.19	10.50	9.77	8.68	12.62	10.98
F-value		36.45	96.43	108.10	17.47	24.82	10.01	15.52	9.17	8.80

*df* Degrees of Freedom, *Cv* Coefficient of variation

^**^ Significant at 5% probability level

#### Effect on cell morphology

**Fig. 5:**
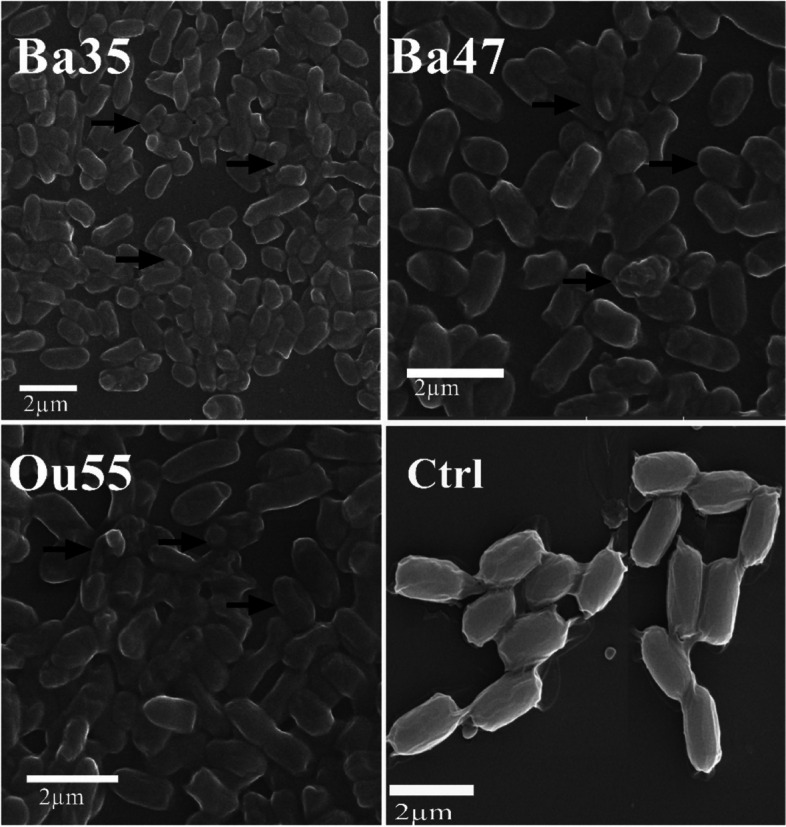
Scanning electron microscopic analysis of the cellular morphology of *A. tumefaciens* Gh1 treated by the CFCS produced by *Pseudomonas kilonensis* Ba35*, Pseudomonas chlororaphis* Ba47, and *Serratia liquefaciens* Ou55 compared to non-treated control (Ctrl)*.* Arrowheads indicated the cell disruption or abnormality

SEM analysis revealed that treating *A. tumefaciens* Gh1 cells to the CFCS generated by Ou55, Ba35, and Ba47 resulted in a broad variety of morphological abnormalities as compared to the non-treated control (Fig. 5). The non-treated control showed normal cell shape and growth. In the presence of the CFCS, however, numerous *A. tumefaciens* Gh1 cells were disrupted and displayed distorted shape.

### *In planta* antibacterial activity of endophytic bacterial suspension against *A. tumefaciens* Gh1

#### Effect of endophytic bacteria on gall weight

**Fig. 6:**
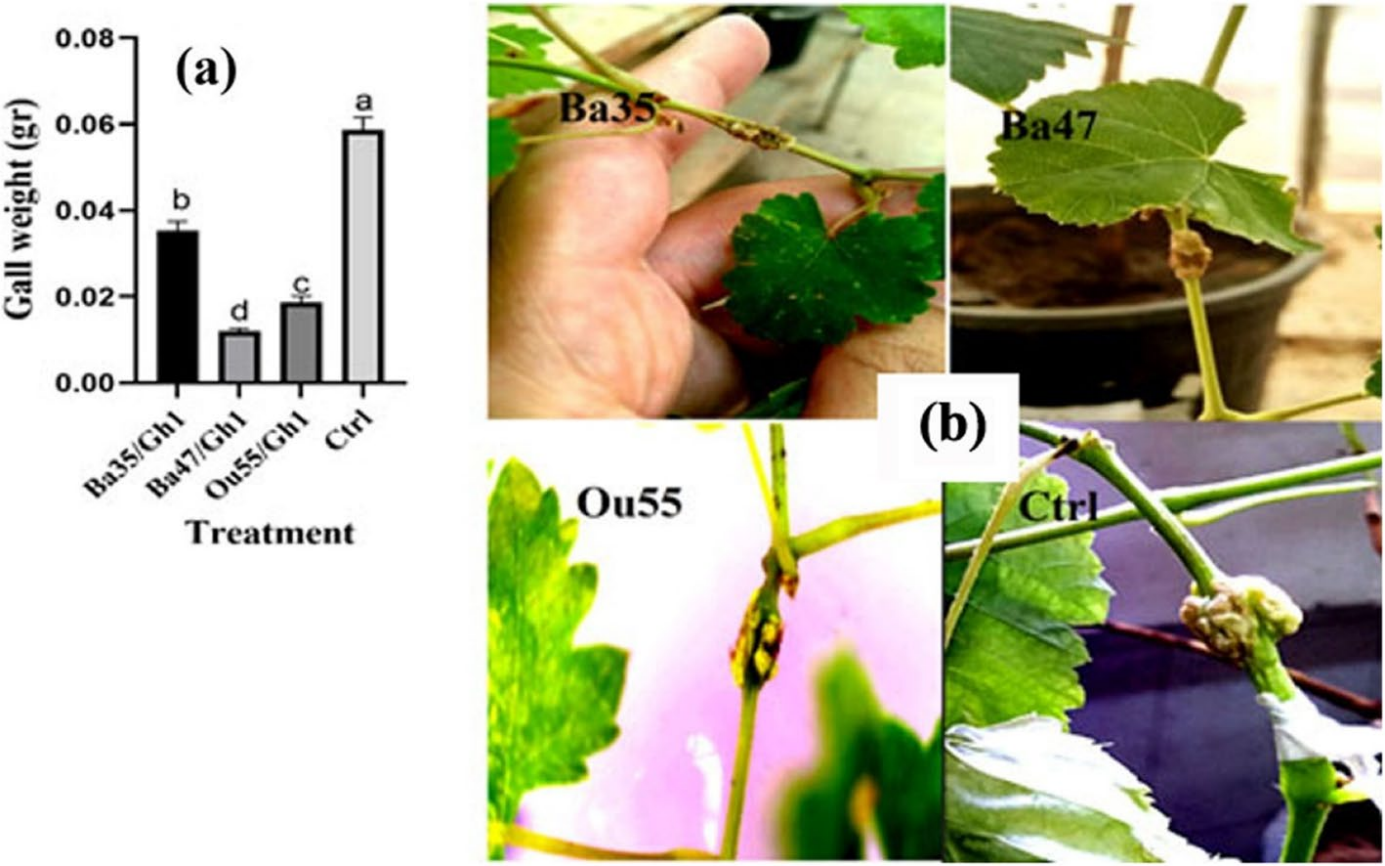
Effect of endophytic bacteria, *Pseudomonas kilonensis* Ba35, *Pseudomonas chlororaphis* Ba47, and *Serratia liquefaciens* Ou55 on gall development by *A. tumefaciens* Gh1 compared to the non-treated control (Ctrl). The comparison of gall weight (**a**), and representative greenhouse assay (**b**) were shown. Graph represent the mean of three replicates. Error bars indicate the SE of three replicates. Different letters indicate significant differences (*P* = 0.05)

Statistically, differences in gall weight decrease across treatments were seen when compared to the control (F = 108.10, P0.0001) (Table 2). The obtained data show that Ba47 had the greatest reducing impact, with about 79.31%, followed by Ou55 and Ba35, with approximately 68.96% and 39.65%, respectively (Fig. 6a &b).

#### Effect on biomass production

**Fig. 7:**
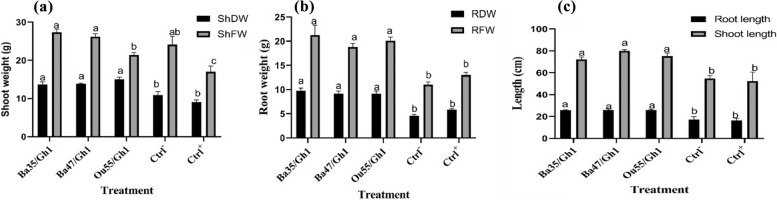
The response of grapevine (*Vitis vinifera* cv. Rashe) 55 days after treated with the endophytic bacteria, compared with non-inoculated (Ctrl-) and *A. tumefaciens* Gh1 inoculated (Ctrl +) plantlets. **a** Shoot dry weight (ShDW) and shoot fresh weight (ShFW), (**b**) Root dry weight (RDW), and root fresh weight (RFW), and (**c**) Root and shoot length. In this experiment the data represent the mean of at least three replicates ± standard error (SE). Column marked by different letters indicate significant differences based on One-way ANOVA, followed by LSD at alpha level = 0.05

Endophytic bacterial strains were tested for their ability to promote plant development in grapevine treated with *A. tumefaciens* Gh1. ANOVA analysis revealed significant differences in root dry weight (F = 24.82, *P* < 0.0001), root fresh weight (F = 17.47, *P* < 0.0001), shoot dry weight (F = 15.52, P0.0001), shoot fresh weight (F = 10.01, *P* < 0.0001), root length (F = 9.17, P0.0001), and shoot length (F = 8.80, *P* < 0.0001) between all treatments (Table 2). In comparison to the non-treated control, plants treated with Ba35/Gh1 and Ba47/Gh1 exhibited greater effects, with fresh shoot weight increases of around 13.21% and 8.59% and shoot dry weight increases of 25.69% and 27.36%, respectively (Fig. 7a). Comparing the three strains to the controls, each one demonstrated a considerable increase in root fresh and dry weight due to the stimulating effects of root development (Fig. 7b). In comparison to the controls, the root length of plantlets treated with strains Ba35/Gh1, Ba47/Gh1, and Ou55/Gh1 increased by 48.34%, 48.68%, and 48.72%, respectively. Comparing the endophytic bacterial treatments to the untreated control, significant differences in shoot length were found. In comparison to plantlets that were infected with Gh1, strains Ba35/Gh1, Ba47/Gh1, and Ou55/Gh1 enhanced the shoot length by 38.22%, 52.87%, and 43.95%, respectively (Fig. 7c).

### Effect on physiology of inoculated grapevine plantlets

#### Effect on total phenolic content (TPC)

**Fig. 8:**
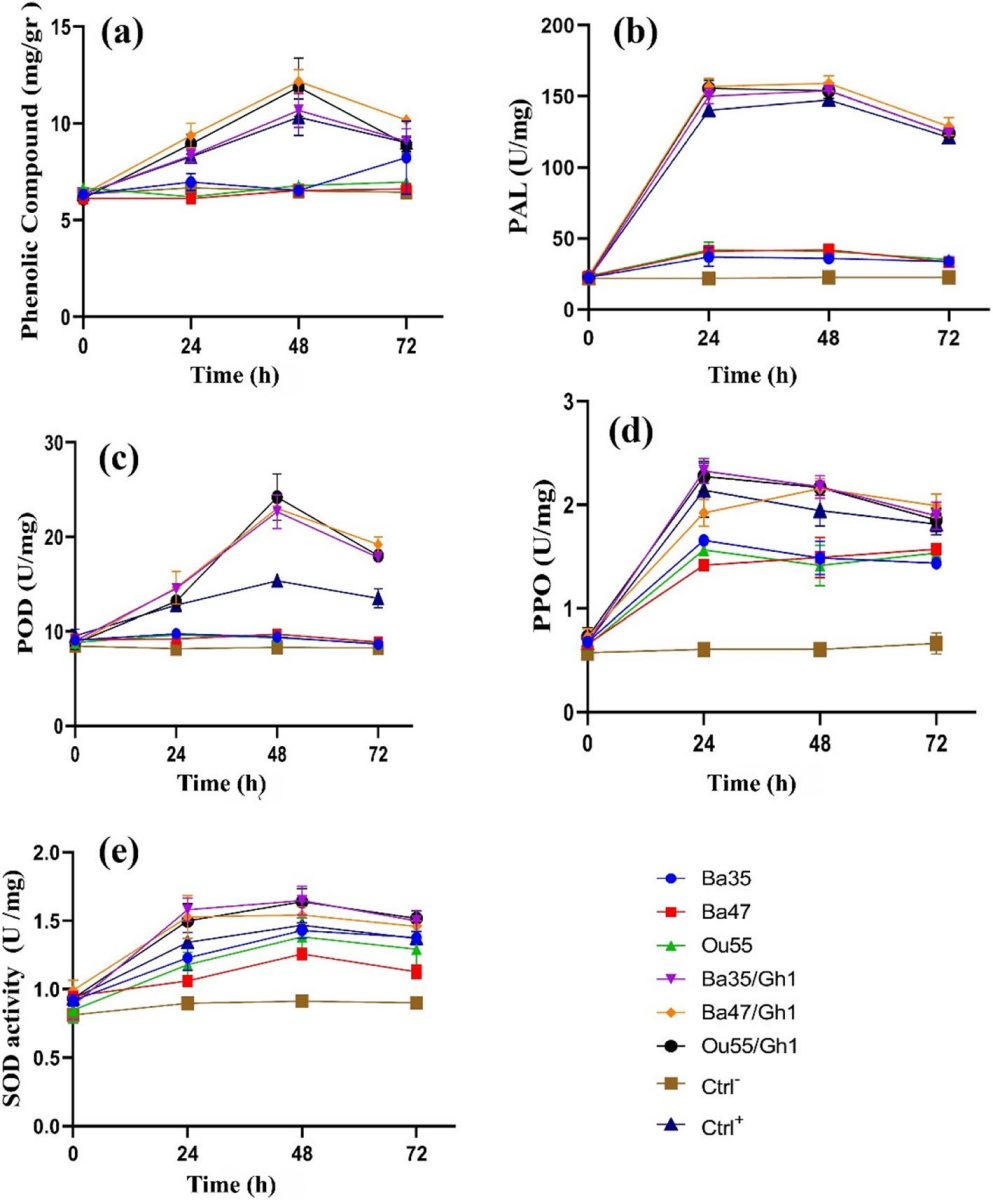
Relative amount of (**a**) total phenolic content, and enzyme activities of (**b**) phenylalanine deaminase, (**c**) peroxidase, (**d**) polyphenol oxidase, and (**e**) superoxide dismutase in the leaves of grapevine plantlets inoculated with *A. tumefaciens* Gh1 pre-treated with the suspension of endophytic bacteria, compared with plantlets inoculated with *A. tumefaciens* Gh1 alone (Ctrl +) and non-treated plantlets (Ctrl-)

The TPC of grapevine plantlet leaves that had been infected with *A. tumefaciens* Gh1 and treated with specific endophytic bacteria were compared to those of non-treated controls, with Gallic acid serving as a reference point (y = 0.002583x-0.008857). Significant differences in the relative defense enzyme activity were seen across all treatments, according to the outcomes of the ANOVA analysis (Supplementary Table 1). When compared to TPC values of the positive control (Ctrl +), TPC in the leaves of plantlets co-treated with Ba47/Gh1 (12.33 + 0.33) and Ou55/Gh1 (12 + 0.58) was considerably greater 48 h after inoculation (Supplementary Table 2 and Fig. 8a).

#### Effect on antioxidant enzymes activity

To determine the amount of defense-related antioxidant enzymes in grapevine plantlets treated with endophytic bacteria and *A. tumefaciens* Gh1, greenhouse experiments were carried out. At four distinct intervals, the enzymatic activity was measured (0, 24, 48, and 72 h). Significant differences in the relative defense enzyme activity were seen across all treatments, according to the ANOVA analysis (Supplementary Table 1). Defense-related enzymes changed in all treatments, and in comparison to the control, the maximal enzymes activity happened at various times.

The mean PAL activity in the leaves of grapevine plantlets co-treated with endophytic bacteria and infected with *A. tumefaciens* Gh1 alone considerably increased when compared to the non-treated control or individual endophytic bacteria alone (Supplementary Table 2). Plantlets infected with Ba47/Gh1 and Ou55/Gh1 had the greatest PAL activity values, whereas Ba35/Gh1 had the lowest values at 24 and 48 h. At 72 h, the PAL activity values began to decline. Nevertheless, at 0 h, no discernible differences were seen between the treatments (Fig. 8b).

After a duration of 48 h, the high POD activity was assessed for all treatments and the positive control (Ctrl +), with the exception of the negative control (Ctrl-). In comparison to the other treatments, co-inoculated plantlets with Ou55/Gh1, Ba35/Gh1, and Ba47/Gh1 exhibited the greatest POD activity. Despite this, there was no discernible distinction between these treatments. At 72 h, the POD enzyme activity decreased gradually in all treatments (Fig. 8c).

Supplementary Table 2 contains the mean PPO enzyme activity in the leaves of grapevine that was inoculated with *A. tumefaciens* Gh1 and treated with endophytic bacteria. The results of the study indicated that grapevine plantlets inoculated with *A. tumefaciens* Gh1, individual endophytic bacteria, or co-treated with endophytic bacteria/pathogen exhibited a substantial increase in their PPO activity in comparison to the non-treated control (Fig. 8d). The plantlets treated with Ba35 (2.3326 + 0.12) and Ou55 (2.27 + 0.15) after being infected with *A. tumefaciens* Gh1 had the highest value of PPO activity after 24 h. However, PPO activity decreased at 48 and 72 h.

Compared to the non-treated control, SOD activity was considerably increased in grapevine plantlets infected with bacterial pathogen alone, individual endophytic bacteria, and co-treatment with endophytic bacteria/pathogen. Treatment of plantlets with endophytic bacteria along with pathogen proved to be even more efficient in increasing the SOD activity as compared with other treatments and control. In comparison to the control, Ba35/Gh1 (1.65 + 0.10), Ou55/Gh1 (1.64 + 0.09), and Ba47/Gh1 (1.54 + 0.06) all had increased SOD activity. At 72 h, the SOD activity gradually decreased in all treatments (Fig. 8e).

#### Expression of defense-related genes

**Fig. 9:**
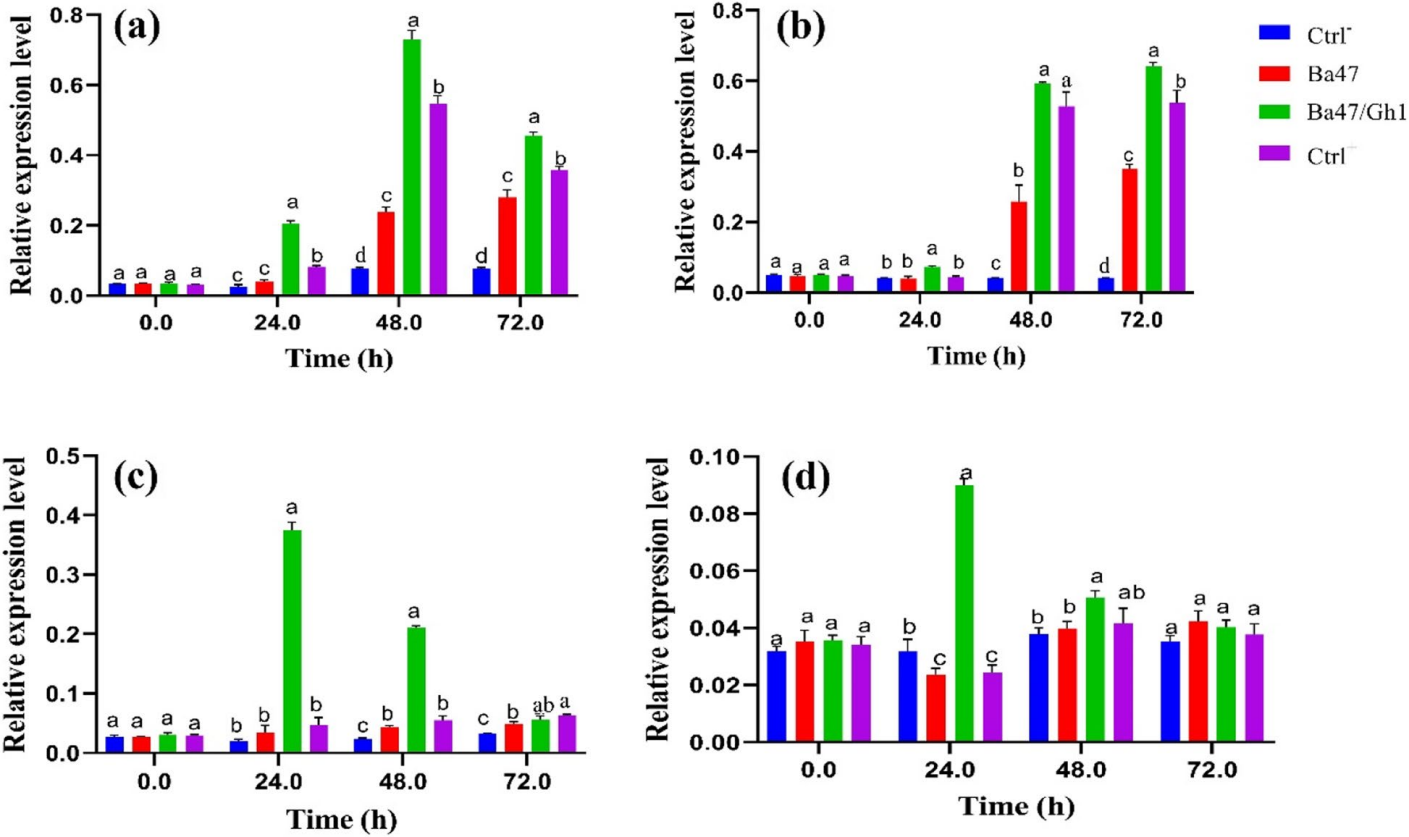
Relative expression levels of *PR1* (**a**)*, PR2* (**b**), *VvACO1* (**c**), and *GAD1* (**d**) genes in the leaves of non-inoculated grapevine plantlets (Ctrl), plantlets treated by *Pseudomonas chlororaphis* Ba47 alone, co-inoculated with Ba47/Gh1 compared with plantlets inoculated with *A. tumefaciens* Gh1 alone (Ctrl +), and non-treated plantlets (Ctrl-). Results represent the means of three replicates. Vertical bars indicate standard errors (SE) and different letters indicate statistically significant differences between treatments at probability levels of 5%

The expression levels of *PR1*, *PR2*, *VvACO1*, and *GAD* in plant samples taken just after inoculation (0 h) remained unchanged for all treatments. The findings demonstrated that, after 48 and 72 h of inoculation, Gh1 alone (Ctrl +) had a much greater impact on gene transcription than Ba47 alone (with the exception of the *GAD* and *VvACO1* genes). In contrast to other treatments, *PR1* gene expression in Ba47/Gh1 was significantly greater in our study at all time periods, with the exception of 0 h following inoculation. At 0 h, there were no discernible differences between the treatments. When compared to controls, the expression of *PR1* in grapevine leaves treated with Ba47/Gh1 rose up to five times at 24 h after inoculation and by about four times at 48 and 72 h (Fig. 9a).

The expression of β-1, 3-glucanase *PR2* gene was induced in grapevine, co-inoculated with Ba47/Gh1 (0.073 + 0.004) was significantly higher compared to controls at 24 h (Fig. 9b).

The expression of *VvACO1* gene was increased in leaves of plantlets treated with Ba47/Gh1 (0.375 + 0.013), and (0.211 + 0.003) at 24 h and 48 h, respectively. The fold-change analysis increased by up to tenfold at 24 h and approximately 5.83-fold at 48 h (Fig. 9c).

*GAD1* gene was significantly induced at 24 h in grapevine leaves treated with Ba47/Gh1 (0.09 + 0.002), although remained induced at 48 h (0.05 + 0.002). At 72 h there was no significant difference among treatments (Fig. 9d).

## Discussion

Crown gall is a soil-borne bacterial disease that may result in significant damage to grapevine plants. In our earlier research, we isolated endophytic bacteria from both cultivated and wild grapevines in Iran with no disease symptoms. Some of these bacteria had the ability to inhibit the crown gall agent [22, 23]. Moreover, the outcomes of the laboratory and *in planta* trials demonstrated that three selected strains (namely, Ba35, Ba47, and Ou55) were able to effectively decrease the symptoms of crown gall in grapevine plants. Hence, these strains possess the capacity to function as biocontrol agents for the management of crown gall disease. The molecular identification results, obtained by analyzing concatenated housekeeping genes, indicate that these bacteria are classified as *Pseudomonas kilonensis*, *Pseudomonas chlororaphis*, and *Serratia liquefaciens*, respectively.

We studied the extent to which these bacteria aided in the prevention of disease and the enhancement of plant development. In sustainable agriculture, CFCS is discussed in a number of reports as a biocontrol agent for bacterial plant pathogens [24]. The antibacterial efficacy of *Bacillus amyloliquefaciens* strain RC-2 culture filtrate against *A. tumefaciens* was shown in a previous work [25]. *Agrobacterium* spp. are biologically controlled by bacteriocin produced by *Bacillus subtilis* strain 14B [26]. Additionally, a bacteriocin found in CFCS of *Bacillus subtilis* IH7 shown bactericidal activity against *A. tumefaciens* [27]. The impact of these CFCS on virulence features of *A. tumefaciens* were examined in this study. It is well known that in order for *A. tumefaciens* cells to be fully pathogenic, they need motility, chemotaxis, and attachment to plant cells [28]. Our research revealed that CFCS of endophytic bacteria might considerably reduce crown gall symptoms by preventing the growth of *A. tumefaciens*. The chemotaxis, motility, biofilm formation, and root attachment of *A. tumefaciens* treated with CFCS of endophytic bacteria are all inhibited in different ways, according to our data.

There is no evidence for other forms of motility, such as swarming and twitching, and previous study revealed that swimming is the most prevalent motility behavior of *A. tumefaciens* [3]. On the other hand, our earlier research shows that *A. tumefaciens* cells have three different motilities including swimming, twitching, and swarming [29]. Towards the plant exudates, *A. tumefaciens* uses its senses and chemotaxis behavior [30, 31]. According to the current study's findings, CFCS of endophytic bacteria may considerably reduce all three types of motility, chemotaxis, and the ensuing grapevine root attachment. This result is consistent with other researches that demonstrated the need of motility and chemotaxis for *A. tumefaciens* attachment [3, 28, 32]. Additionally, the *A. tumefaciens* Gh1 cell population was decreased by the CFCS of endophytic bacteria. As a consequence, our findings suggest that CFCS may oppose *A. tumefaciens* via a variety of antagonistic ways.

*A. tumefaciens* has been shown to be able to form biofilm on plant surfaces that aids in plant tissue adhesion [33]. Moreover, surface attachment and biofilm formation are lacking in non-motile cells [3]. Finding of the present study show a clear correlation between the reduction effect of CFCS on motility and biofilm development of *A. tumefaciens* cells, as well as their root attachment. In the current study, the attachment of CFCS-treated *A. tumefaciens* cells to grapevine cv. Rashe was examined in both wounded and unwounded roots, and the results were contrasted with those of the untreated control. The obtained findings demonstrated that *A. tumefaciens* cell attachment to the grapevine root was greatly inhibited by CFCS. Furthermore, no significant distinctions were found between the attachment to wounded and unwounded grapevine roots. This result is in line with the earlier study that shown *A. tumefaciens* bv.1 attached to both damaged and unwounded grape roots equally [32].

In grapevine infected with *A. tumefaciens* Gh1, endophytic bacterial strains were assessed for their capacity to promote plant development. According to the findings, in comparison to the controls, all three strains exhibited stimulating effects on shoot and root development. The findings demonstrated that endophytic bacteria could raise grapevine biomass even when there was no special treatment for them. *Serratia* and *Pseudomonas* are two genera of endophytic bacteria that have been reported to positively impact plant development [34].

Plants that are resistant to disease have defensive mechanisms activated, which may lower or stop infection at certain phases of the host–pathogen relationship. The coevolution of endophytic bacteria with plants enables them to establish a mutually beneficial and stable connection with the related plants [35]. This research further seeks to evaluate the impact of endophytic bacteria on the interactions between *A. tumefaciens* and grapevines, with a specific emphasis on the regulation of defense enzymes.

The oxidative burst is an initial and rapid reaction of plants to pathogen infection, resulting in the production of a substantial quantity of reactive oxygen species (ROS) as a defensive response. Different non-enzyme compounds, such as phenol compounds, and antioxidant enzymes, such POD, PPO, PAL, and SOD, may scavenge ROS [36]. Additional research revealed that during plant–microbe interactions, both pathogenic and helpful bacteria raise the levels of ROS and phenolic compounds in plant hosts [37]. Findings of the present study indicate that, in comparison to other treatments and the untreated control, the phenolic content rose considerably after treatments with Ba35/Gh1, Ba47/GH1, Ou55/Gh1, and Gh1 alone. This result is consistent with earlier research that demonstrated the production of antioxidant enzymes and phenolic compounds in tomato plants infected with *A. tumefaciens* after pretreatment with strains of *Bacillus* and *Pseudomonas* [38]. Under conditions of stress and pathogen infection, phenolic chemicals are often generated and accumulate in the plant tissues. They serve as defensive systems for plants against bacterial and fungal infections [31]. Phenolic chemicals are known to have a function in chemotaxis and to promote the expression of virulence genes during the interaction between *Agrobacterium* and plant [39].

Changes in plant cell metabolism are brought about by the interaction of the pathogen with the host plant, especially in the activity of defense-related enzymes such POD, PPO, PAL, and SOD. POD is a crucial enzyme that takes part in the formation of lignin and other activities involving plant cell walls. PPO is also known to accelerate the oxidation of phenolic substances to free radicals, which may react with biological molecules and create an environment that is detrimental to the growth of pathogens. PAL is a crucial enzyme that is involved in the biosynthesis of secondary chemicals associated to defense, such lignin and phenols. The first enzyme in antioxidant defense is SOD.

According to the finding of the current study after 24 h, co-inoculation of grapevine plantlets with pathogen and endophytic bacteria revealed significant levels of total phenol concentrations and antioxidant enzyme activity. In plant samples taken just after inoculation, no discernible differences were seen (0 h). The results demonstrated that, as compared to grapevine plantlets that were not treated, POD, PPO, SOD, and PAL activities were much greater in those that had been co-inoculated with Ba47/Gh1, Ou55/Gh1, Ba35/Gh1, and Gh1 alone. These findings likely indicate that the host defense system has been activated. Plants treated alone with endophytic bacteria have increased PPO and SOD activities. However, there is no observed increase in total phenolic compounds, PAL, and POD activities. This finding is corroborated by prior studies that indicated that treatment of plant with antagonists may lead to increased levels of these enzymes after pathogen infection [40]. The antioxidant enzyme activities and total phenolic compounds in the leaves of the control plants were only at baseline levels, and there was no discernible difference in the specific activity of these compounds.

These findings are consistent with earlier research showing that a considerable rise in ROS levels after *A. tumefaciens* infection is probably linked to plant resistance [41]. Similar findings shown that *Pseudomonas syringae* pv. *phaseolicola* significantly increased the activity of antioxidant enzymes including SOD and POD [42]. Consistent with our findings, earlier research shown that these enzymes' activity was markedly elevated in plants infected with *Ralstonia solanacearum*, the plant pathogen that causes bacterial wilt [43]. Additionally, prior research has shown that in transgenic tomatoes, an overexpression of PPO enzyme activity results in bacterial disease resistance [44]. According to a different research, resistance of potato to *Pectobacterium atrosepticum*, *P. carotovorum* subsp. *brasiliensis*, and *Dickeya* spp., bacterial soft rot agents, is influenced by PAL, PPO, POD, and total soluble phenols [45]. Activating the expression of defensive response genes is the second way that endophytic bacteria might induce the resistance response in infected plants. According to our earlier research, grapevine plantlets treated with *Pseudomonas* sp. Sn48 and *Pantoea* sp. Sa14 showed considerably higher levels of *PR1*, *PR2*, and *PR4* gene expression after *A. tumefaciens* inoculation [23]. Additional studies on the interactions between fungal infections and/or abiotic stressors and beneficial bacteria that develop systemic resistance in grapevine have been conducted [46–51]. In this work, we examined the expression of *ACO*, *GAD*, and *PR2* which codes for ACC oxidase, glutamate decarboxylase, and β-1,3-glucanase respectively, and *PR1*, a marker of the salicylic acid pathway, in leaves after Ba47 treatment and *A. tumefaciens* inoculation.

According to the present study's findings, leaves taken from grapevine plantlets that had been treated with endophytic bacteria expressed more *PR1* and *PR2* genes. According to our findings, *A. tumefaciens* Gh1 significantly increases the induction of *PR1* and *PR2* transcription compared to endophytic bacteria, and Gh1 is primarily responsible for the significant increase in transcription that occurs after co-inoculation with Ba47/Gh1. A previous research found that elevated enzyme activities and PR-protein levels are responsible for the control of the bacterial blight disease caused by *Xanthomonas oryzae* [52]. Additional research revealed that *PR2* could regulate the defensive responses against bacterial disease that are reliant on callose and SA [53].

Previous studies showed that owing to the effective colonization of endophytic bacteria, plant tissues either did not react or responded poorly to helpful bacteria, resulting in the reducing activation of defensive responses [54]. When compared to the untreated control, the leaves obtained from plantlets treated with Ba47 alone and after an *A. tumefaciens* challenge had higher levels of *PR1* and *PR2* genes. Consistent with our findings, earlier research has shown that *A. tumefaciens* infection induces a number of defensive mechanisms in plants, including increased expression of *PR1* and *PR2* genes [23, 55].

One of the essential parts of ethylene production is 1-aminocyclopropane-1-carboxylic acid oxidase (ACO), which is encoded by the multigene family *VvACO1*. In addition to acting as a stress signal for plants, ethylene is important for defensive mechanisms against pathogen invasion. According to earlier research, ethylene has a significant role in regulating the pathogenicity of *Agrobacterium, according* [56]. Numerous plant-associated bacteria have the ability to alter the quantities of ethylene and ACO in plants, which in turn may alter the way that plants respond to biotic stress [18]. According to our findings, following 24 and 48 h of treatment with Ba47, *A. tumefaciens* Gh1, and Ba47/Gh1, the expression of the *VvACO1* gene was considerably higher in the leaves collected from grapevine plantlets than in the untreated control. Our findings support a prior study that found ethylene levels were up-regulated during *Agrobacterium*-plant interaction [57]. The Ba47/Gh1 treatment showed the greatest amount of *VvACO1* gene expression. These findings imply that ethylene affects the interactions between *Agrobacterium* and plant, at least partly, by reducing the virulence of the bacterium.

GABA is a crucial molecule that plays a part in plant defense and is produced in plants by the *GAD* operon [58]. Previous research found that GABA promotes the deactivation of the *Agrobacterium* quorum-sensing signal in infected plants. Moreover, plants that have greater levels of GABA are less susceptible to infection by *A. tumefaciens* [59]. Findings of the present study indicate that, in comparison to other treatments and the control, the *GAD1* gene expression level was considerably higher in leaves harvested from plantlets treated with Ba47/Gh1 after 24 h. Inhibition of symptoms associated with crown gall disease in Ba47/Gh1 treatment may be correlated with an increase in GABA levels. However, this is genetic evidence, and additional research is required to ascertain the precise concentration of GABA in plantlets treated with endophytic bacteria during pathogen infection.

## Conclusions

This is the first study on how *A. tumefaciens*, the grapevine crown gall agent, is affected by CFCS generated by endophytic bacteria in terms of growth and virulence features. Our findings showed that these endophytic bacteria inhibit the growth of bacterial pathogen and limit their ability to invade plant roots and/or develop resistance in grapevines. Based on the results of this investigation, we draw the conclusion that endophytic bacterial suspension-induced defense-related enzymes such PAL, POD, PPO, and SOD may shield the plant from infection. In grapevine leaves, the activation of *PR1*, *PR2*, *VvACO1*, and *GAD1* genes implies that the SA and JA pathways are involved in the development of resistance to pathogen infection. According to our research, treating grapevines with the Ba47 strain increased the expression levels of defense-related genes *PR1*, *PR2*, *VvACO1*, and *GAD1*, which most likely contributed to the induction of systemic resistance to the agent causing crown gall disease. Since *A. tumefaciens* is a soilborne pathogen, adding these endophytic bacteria to the soil may be a helpful way to boost plant development and lower the incidence of crown galls. To create safer plant disease management techniques, knowledge of the biocontrol processes mediated by endophytic bacteria during microbial interactions is crucial. The antibacterial compounds in these CFCS have not been documented, but further research is needed. There has not been much research done on the cytotoxicity of CFCS on plant tissues. Thus, more research on the safety and cytotoxic effects of CFCS is also required. To determine if these endophytic bacteria are potential biocontrol agents, further field research is required.

## Materials and methods

### Bacterial strains

The endophytic bacteria Ba35 (*16S rRNA* GenBank Acc. No. MK114598), Ba47 (*16S rRNA* GenBank Acc. No. MK114597), and Ou55 (*16S rRNA* GenBank Acc. No. MK114620), previously isolated from wild growing and domesticated grape, as well as *A. tumefaciens* Gh1 (GenBank Acc. No. MK114594) which showed crown gall disease in grapevine [22] were used in this study. Bacteria were cultured in liquid Lauria-Bertani (LB) or nutrient agar (NA) medium for 24 h at 26–28 °C with 150 rpm shaking. Bacteria were collected by centrifugation and suspended in distilled nutrient broth (NB) medium, concentration was subsequently adjusted to approximately 10^8^ and 10^6^ CFU/ml for endophytic bacteria and *A. tumefaciens* respectively, and stored at -20 °C.

### Molecular identification of endophytic bacteria

Endophytic bacteria were further identified by multilocus sequence analysis (MLSA) due to the concatenation of two housekeeping genes (*16S rRNA* and *rpoD*) for *Pseudomonas* strains*,* and *16S rRNA* and *pgi* genes for *Serratia* strain. *Pseudomonas* strains were identified by partial nucleotide sequencing of the *rpoD* gene using primers PsEG30F (5'- ATYGAAATCGCCAARCG-3') /PsEG790R (5'-CGGTTGATKTCCTTGA-3') based on the method previously described [60]. The amplification conditions were as follows: five minutes of denaturation period at 94 °C, followed by 30 cycles of amplification (denaturation at 94 °C for 1 min, annealing at 55 °C for 1 min, and extension at 72 °C for 1.5 min). A final extension step was done at 72 °C for 10 min. *Serratia* strain was identified by partial nucleotide sequencing of the *pgi* gene applying PCR with primers pgiF (5'-TCT YTI GGI TTT GAK AAY TTT GA-3')/ pgiR (5'-YGC CGC YGI AAA TTC IGC TTC-3') [61]. The amplification conditions included a denaturation at 95 °C for 3 min, followed by 30 cycles of denaturation at 94 °C for 30 s, annealing at 52 °C for 30 s, extension at 72 °C for 1 min, and a final extension at 72 °C for 10 min.

An ABI3730XL DNA sequencer (Applied Biosystems) was used to sequence the PCR products. Using the BioEdit sequence alignment editor 7.0.9.0 program, the acquired sequences were aligned and manually modified [62]. Using the BlastN tool, the *rpoD* and *pgi* gene sequences were further subjected to BLAST analysis against additional sequences that had been obtained from the NCBI database. Using MEGA version 11.0, the maximum likelihood phylogenetic analysis was carried out, and a phylogenetic tree was created (bootstrap analysis with 1000 repeats was conducted) [63].

### Plant materials, and growth conditions

Nodal explants grown on MS media [64] were used to micropropagate plantlets of *Vitis vinifera* cv. Rashe, as previously reported with a few changes [65]. After collecting a few of the stem's buds and gently shaking them in 70% ethanol for a minute and hypochlorite for four minutes, the meristems were separated and placed in tubes with 1/2 MS media for a period of two weeks. Following disinfection, the explants were moved to jars with MS media and kept for four weeks at 25 ± 2 °C. The explants were kept in the growth chamber at 25 ± 2 °C for 16 h of light and 8 h of darkness after sub-culturing. Lastly, grapevine plantlets were planted in pots filled with steam-sterilized soil (pH 7.2, 50% sand, 20% clay, 30% peat), and kept in a greenhouse at 25–26 °C, 16 h of day and 8 h of night, and 95% relative humidity.

### Antibacterial activity of CFCS produced by endophytic bacteria

After culturing endophytic bacteria in 5 ml LB medium at 26–28 °C until the final concentration reached around 1 × 10^8^ CFU/ml, the cultures were centrifuged for 10 min at 13,000 × g. The supernatants were collected and sterilized using 0.22 μm filters to achieve CFCS.

Using the agar diffusion technique, the antibacterial activity of CFCS generated by endophytic bacteria against *A. tumefaciens* Gh1 was evaluated. Nutrient agar media was prepared, after making a hole with a sterile Cork borer that measured 5 mm in diameter and 2–3 mm in depth, 10 μl of CFCS was added. Following an overnight incubation period, a 10 μl bacterial pathogen suspension (1 × 107 CFU/ml) was spread onto the medium and allowed to remain at room temperature for five minutes. After that, the plates were maintained at 26–28 °C for 48–72 h, during which time the width of the growth inhibition zone was measured [66]. For every treatment, three replications were carried out.

### Mode of action of CFCS against *A. tumefaciens*

#### Effect on swarming, swimming, and twitching motility

Surveys were conducted on the motility characteristics of *A. tumefaciens* Gh1 cells treated with endophytic bacteria-derived CFCS. *A. tumefaciens* Gh1 was grown overnight and its concentration was roughly adjusted to OD_600_≃0.8. *A. tumefaciens* Gh1 cells (40 μl) were combined with 160 μl CFCS and incubated for 24 h at room temperature. Subsequently, two microliters were added to LB medium supplemented with agar (0.2, 0.7, and 1.6%), for the purposes of swimming, swarming, and twitching motility, respectively. The plates were incubated at 26–28 °C, and after 48 and 72 h, the motility halo diameter was determined. Three replications of the experiment were carried out.

#### Effect on chemotaxis

A chemotaxis buffer medium was prepared (0.1 mM EDTA, 10 mM K2HPO4, 0.35% agar, pH 7.2). After removing 5 mm in diameter of the medium, refilled it with 50 μl of grapevine root extract (produced by homogenizing 1 g of grape roots in 10 ml of sterile 0.1 M phosphate buffer at 150 rpm for 24 h and sterilizing with 45 μm filter paper). After allowing 40 μl of *A. tumefaciens* Gh1 cells (OD_600_≃0.8) treated with 160 μl CFCS to remain at room temperature for 24 h, a spot inoculum of 5 μl of the mixture was spot inoculated 5 mm away from the hole. After being covered with parafilm, the plates were allowed to maintain at room temperature. The CFU/ml measurement represented the migration of the bacterial pathogen cells toward the root extract. As a control, *A. tumefaciens* Gh1 cells that had not been treated with CFCS were used. Three replications of the experiment were carried out.

#### Effect on biofilm formation

In polypropylene tubes, the biofilm production of *A. tumefaciens* Gh1 cells treated with CFCS was evaluated. In summary, endophytic bacteria were growth for 48 h at 26–28 °C in LB medium. Centrifugation (10 min, 6000 rpm) and filtration were used to extract CFCS, which was then verified by incubating 100 μl of supernatants on LB agar confirming by no bacterial growth. 40 μl of *A. tumefaciens* Gh1 cells (OD_600_ ≃ 0.8) were combined with 160 μl of CFCS and incubated at 26–28 °C for 24 h without being shaken. Each microtube was filled with 5 μl of 1% crystal violet solution, which was let to maintain at room temperature for 15 min. After that, sterile water was used twice to rinse the microtubes. After adding 2 × 200 μl of 95% ethanol to each tube, the total volume was raised to 1 ml with sterile-distilled water, and a spectrophotometer (SPECORD 210, Analytik Jena, Germany) was used to measure the absorbance at 540 nm. As a control, *A. tumefaciens* Gh1 cells were not treated with CFCS. The experiment was carried out in three replications using a fully randomized design [67].

#### Effect on grapevine root attachment

Following a 48-h treatment with CFCS, the attachment of bacterial pathogen cells to both injured and unwounded roots of grapevine plantlets was examined. In short, the roots were stored at room temperature in 10 ml treated or untreated bacterial pathogen cell suspensions (adjusted to about 1 × 108 CFU/ml) and were washed three times with sterile distilled water after three hours. Next, the tips of the roots were separated by 3–5 mm, weighed, and then each piece was immersed in 1 ml of sterile-distilled water. The roots were macerated in 100 ml of sterile water after being stirred for 5 s. After streaking the resulting suspension over NA media and incubating it for 48 h at 28 °C, the CFU/ml was measured. Three replications and a fully randomized design were used to carry out the experiment [68].

#### Effect on cell morphology

External morphological changes of bacterial pathogen cells were observed using scanning electron microscopy (SEM). Bacterial cells were placed into Eppendorf tubes and washed twice with 0.1 M phosphate buffer saline (PBS, pH:7.2) before being distributed on a clean slide with or without being treated with the CFCS for 72 h at 26–28 °C. Following a one-hour fixation in a 2.5% glutaraldehyde solution at room temperature, the samples underwent three PBS washes. Samples were dehydrated by ethanol solutions containing 30, 40, 50, 60, 70, 80, and 96% for 15 min each time, followed by 96% ethanol for 1 h. After that, the samples were freeze-dried for three hours at -40 °C. After applying a gold coating to the samples, an electron microscope (Philips SEM, Netherlands) was used to capture electron micrographs.

#### Effect of endophytic bacteria on crown gall disease development

Endophytic bacteria were tested *in planta* to determine their antagonistic activity against *A. tumefaciens*. Endophytic and bacterial pathogens were cultured in LB medium at 26–28 °C for 24 h before being suspended in sterile-distilled water with a density of OD_600_≃1.0. One ml suspension of endophytic bacteria was given to the pots containing plantlets (prepared as previously mentioned in the section on plant materials and growth conditions). Following a week, a sterile toothpick was used to puncture the stems, and a sterile syringe was used to inoculate 20 μl of the bacterial pathogen between the third and fourth internodes. Inoculated plantlets were maintained in a greenhouse with temperature of 25–26 °C, a 95% humidity level, and a 16–8 h day/night photoperiod. Up to thirty days of gall production records were kept, and the weight of the new galls was calculated. The positive and negative controls were inoculated plantlets with the pathogen alone or sterile water, respectively. Notably, a fully randomized design (CRD) was used to evaluate each treatment on three distinct grapevine plantlets.

#### Effect of endophytic bacteria on grapevine biomass production

The biomass output of grapevine plantlets that had been inoculated with *A. tumefaciens* Gh1 and pre-treated with endophytic bacteria (as mentioned in the section on plant materials and growth conditions) was assessed. After being inoculated, the plantlets were collected five weeks later. Following their separation and three rounds of rinsing in sterile distilled water, the fresh weight and length of each treatment's root and shoot were measured. Following a 48-h drying period at 50 °C, the dry weight of the shoot and root was determined. For every treatment, three replicates were assessed.

Additionally, leaves from all treatments were taken at 0 h, 24, 48, and 72 h following *A. tumefaciens* Gh1 inoculation, and they were kept at -20 °C for analysis of defense-related gene expressions, quantification of total phenolic content, and measurement of antioxidant enzyme activities.

### Effect of endophytic bacteria on the physiology of inoculated grapevine plantlets

#### Effect on total phenolic content (TPC)

The total phenolic content was measured according to the Folin- Ciocalteau method previously described [69]. One gram of fresh leaves was crushed in a mortar with 10 ml of 80% methanol, then centrifuged at 10,000 g for 15 min [70]. Next, 5 ml of sterile-distilled water were mixed with 1 ml of the crude methanol extract and 250 μl of Folin–Ciocalteau reagent (Sigma-Aldrich, Germany). After five minutes incubation at 25 °C, 1 ml of 20% Na_2_CO_3_ was added to the solution. The spectrophotometer SHIMADZU 1800 UV was used to measure the absorbance value at 765 nm after the solution had been left at room temperature for two hours. Gallic acid (GA) was used as the standard in a calibration curve that was created utilizing the same laboratory setup that was used for the *Vitis vinifera* plant analysis. The result was given in GA equivalents (μg/ml). Every analysis was carried out three times.

#### Effect on induction of antioxidant enzymes

Antioxidant enzyme activity was measured in leaves taken from grapevine plantlets treated with endophytic bacteria and *A. tumefaciens* Gh1 (as described in section plant materials and growth conditions). Superoxide dismutase (SOD), phenylalanine deaminase (PAL), polyphenol oxidase (PPO), and peroxidase (POD) were among these enzymes. Leaf samples from the grapevine cv. Rashe were taken at various times (0, 24, 48, and 72 h) in order to extract the enzymes. At each time interval, three to five leaf samples were randomly selected, wrapped in aluminum foil, rapidly submerged in liquid nitrogen, and stored at -80 °C until needed.

Leaf tissue weighing about 0.2 g was crushed in liquid nitrogen, homogenized with 2 ml of extraction buffer (0.1 M phosphoric acid buffer, pH 7.8 plus polyethylene pyrrolidone) that had been pre-cooled, and centrifuged for 20 min (4 °C at 13,000 rpm). The supernatant was poured into a fresh tube, 3 ml of extraction buffer was added, and after a 1-h room temperature incubation, the mixture was centrifuged for 30 min (40 °C at 12,000 rpm). Then, superoxide dismutase (SOD), peroxidase (POD), phenylalanine deaminase (PAL), and polyphenol oxidase (PPO) were measured using the supernatant.

The previously described approach was used to record the PPO activity [71]. The reaction mixture included sodium phosphate (0.1 M) buffer (pH 7.4), 3.0 ml of substrate solution with catechol (0.1 M) as the substrate, and 0.1 ml of protein extract. In the control, the extract was replaced with 1 ml of sodium phosphate buffer. For one minute at 25 °C, the rate of catechol oxidation was measured at 495 nm. The rise in absorbance by 0.001 min was used to determine the enzyme activity.

The measurement of POD activity included the oxidation of a 0.1 ml protein sample in a mixture consisting of 3 ml of 0.05 M phosphoric acid buffer (pH 5.5), 2 ml of 2% hydrogen peroxide (H_2_O_2_) as the oxidizing agent, and 36 μl of guaiacol as the hydrogen donor per 50 ml of the buffer solution. The guaiacol oxidation was performed at a wavelength of 470 nm, at a temperature of 25 °C. The results were represented in units per gram (U/g) of fresh weight per minute (FW/min). Enzyme activity was determined by measuring the rate of rise in absorbance of 1 unit of enzyme at 470 nm per minute at a temperature of 25 °C. The activity of POD was quantified as U mg/protein [71]. The enzyme activity was calculated using the following formula:

$$POD\;Vigor\;\left(U\;g^{-1}fw\;{min}\hat{\phantom{i}}-1\right)=\frac{\lbrack(a\hat{\phantom{i}}(initial-a\hat{\phantom{i}}2min)\times enzyme\;Liquid\;total\;volume\;(ml))\rbrack}{\lbrack2(\times sample\;quality\;(g)\rbrack}$$

With a few modifications, the prior method was used to assess PAL activity [71]. 0.1 ml of protein extract, 0.1 M phosphoric acid buffer (pH 8.8), and 1.0 ml of 20 mM l-phenylalanine made up the test mixture. At 37 °C, the mixture was incubated for 30 min. The addition of 0.1 ml of HCl (0.6 M) terminated the reaction. The synthesis of trans-cinnamate served as the basis for determining PAL activity. The product of trans-cinnamic acid was separated using 5 ml of ethyl acetate. Three ml of 0.05 mol/l NaOH were used to dissolve the solid residue after evaporation of extracting solvent. In order to calculate the concentration of cinnamic acid, the absorbance was measured at 290 nm. The crude protein combined with l-phenylalanine without any incubation period was represented as the blank.

$$Pal\;Vigor\;\left({Ug}^{-1}fw-1\right)=\frac{\lbrack a^{290}\times\;total\;volume\;of\;enzyme\;liquid\;\left(ml\right)\rbrack}{\lbrack30\;\left(ml\right)\;\times\;sample\;quality\;\left(g\right)\rbrack}$$

The procedure outlined in [71] was used to record the estimate of SOD activity. 50 mM potassium phosphate (pH 7.8), 14.5 M d-methionine, 2.5 mM NBT, 3 μM EDTA, and 60 μM riboflavin were combined to prepare a reaction mixture. The tubes holding 0.1 and 1.0 ml of protein extract and reaction media, respectively, were placed under the 20 W fluorescent light for 15 min. The absorbance was measured using a spectrophotometer at 560 nm. The quantity of enzyme producing 50% inhibition of photochemical reduction of NBT was defined as one unit (U) of SOD activity. The SOD activity was expressed in U/mg of protein and determined by using the following formula:

$$SOD\;vigor\;\left(U\;g^{-1}\;fw\right)=\frac{\lbrack\left(Control-Sample\right)\times100\%\times total\;volume\;of\;enzyme\;liquid\;\left(ml\right)\rbrack}{\lbrack Control\times50\times0.1\;\left(ml\right)\times Sample\;quality\left(g\right)\rbrack}$$

The blank solution was stored in the dark, and the reaction medium without a protein sample served as the control.

### Expression of defense-related genes against *A. tumefaciens* Gh1

In the leaves of grapevine cv. Rashe, the expression of target genes, such as *PR1*, *PR2*, *VvACO1*, and *GAD1*, as indicators of the pathways involved in the biosynthesis of ethylene and salicylic acid (SA) respectively, was assessed using qRT-PCR. Plantlets treated with *A. tumefaciens* Gh1, *A. tumefaciens* Gh1/Ba47, and Ba47 (which displayed decreased gall weight) as well as untreated plantlets' leaves (as a negative control) were collected after 0,24,48, and 72 h, wrapped in aluminum foil, and kept in a sterile microtube at -80 °C until needed. 2 g of frozen, powdered tissues were macerated in 2 ml of extraction buffer (13 ml of saturated phenol, 0.32 ml of sodium acetate, 0.01 ml of EDTA, 1% SDS, and 1% PVP per 15 ml of TE buffer pH: 8.0) in order to extract RNA. After centrifuging the suspension for five minutes at 8,000 rpm and 4 °C, the supernatant was poured into a fresh tube. After centrifuging the mixture containing the supernatant and equal volumes of phenol, chloroform, and isoamyl alcohol (25/24/1), RNA was precipitated using 0.1 volume of 3 M sodium acetate (pH: 5.0) and an equal amount of isopropanol, which was left overnight at 20 °C. Ultimately, the suspension was centrifuged for 15 min at 13,500 rpm. After air drying and a 70% ethanol wash, RNA was dissolved in 50 ml of RNase-free DEPC water. Using NanoDrop, the concentration and purity of RNA were measured (Thermo Fisher Scientific, United States). DNase1 (Yekta Tajhiz Azma, Iran) was used to remove the DNA contamination, and RNA content was determined at 260 nm.

Using a random hexamer primer and the cDNA synthesis kit (Parstous, Iran) according to the manufacturer's instructions, first-strand complementary DNA (cDNA) was synthesized using 200 ng of RNA as a template. In a StepOne thermal cycler (AB Applied Biosystem, USA), real-time PCR was carried out using a 12 μl reaction mixture that included 6 μl master mix green high ROX, 1 μl of cDNA, 0.5 μM of each gene-specific primer (Table 3), and 4 μl DEPC-treated water. The reference gene used was the translation elongation factor 1 alpha (*EF-1 alpha*) gene. 95 °C (15 min), 40 cycles of 95 °C (15 s), 60 °C (30 s), and 60 °C (1 min) comprised the PCR procedure. Using the comparative CT approach (ΔCT method), primer sufficiency was verified and the relative gene expression was calculated in relation to the *EF-1 alpha* gene [72].

**Table 3:** Primer sequences used in RT-PCR

Gene target	Primer sequences	Reference
*PR1*	Forward: 5'-GGAGTCCATTAGCACTCCTTTG-3' Reverse: 5'-CATAATTCTGGGCGTAGGCAG-3'	[50]
*PR2*	Forward: 5'-TCAATGGCTGCAATGGTGC-3' Reverse: 5'-CGGTCGATGTTGCGAGATTTA-3'	[50]
*GAD1*	Forward: 5'-GGTCCTCCGAGGCGATAATG-3' Reverse: 5'-CTCCCAGCACACCTGAACAT-3'	This study
*VvACO1*	Forward: 5'-CGCCCAAACTGATGGAAACAGAATG-3' Reverse: 5'-GGGTACACTTCGCTTGTCTCCTTT-3'	[73]
*EF1*	Forward: 5'-AACCAAAATATCCGGAGTAAAAGA-3' Reverse: 5'-GAACTGGGTGCTTGATAGGC-3'	[50]

Relative gene expression was calculated applying the following formula [74].

$$\mathrm{Relative}\;\mathrm{expression}=\text{e}^{-\mathrm\Delta\text{ct}}=\text{e}^{-\left(\mathrm{Ct}\;\mathrm{target}\;\mathrm{gene}-\mathrm{Ct}\;\mathrm{reference}\;\mathrm{gene}\right)}$$

Ct values were the means of three biological and three technical replications.

### Statistical analysis

Data were analyzed by the analysis of variance (ANOVA), followed by the Least-Significant Difference (LSD) test (*P* = 0.05), applying the SAS (version 9.1) program. All the experiments were conducted in a completely randomized design. Graphs and figures were plotted using Sigma plot, Minitab, and GraphPad Prism program.

### Supplementary Information

**Additional file 1: Supplementary Table 1.** Analysis of variance (ANOVA) of relative defense enzymes activity in leaves of grapevine plantlets inoculated with endophytic bacteria and *A. tumefaciens* Gh1 after 0, 24, 48, and 72 h. **Supplementary Table 2.** Relative defense enzymes activity in leaves of grapevine after endophytic bacteria and *A. tumefaciens* Gh1 inoculation.

## Data Availability

All gene sequence data and bacterial strains information are deposited in NCBI database and publicly available through the web link: https://www.ncbi.nlm.nih.gov/nuccore/ MK114594, https://www.ncbi.nlm.nih.gov/nuccore/ MK114597, https://www.ncbi.nlm.nih.gov/nuccore/ MK114598, https://www.ncbi.nlm.nih.gov/nuccore/ MK114620, https://www.ncbi.nlm.nih.gov/nuccore/ OQ657168, https://www.ncbi.nlm.nih.gov/nuccore/ OQ657169, https://www.ncbi.nlm.nih.gov/nuccore/ OQ657170. Other data that support the findings of this study are available from the corresponding author upon reasonable request.
