# Supplementary material for: Antivirulence effects of cell-free culture supernatant of endophytic bacteria against grapevine crown gall agent, Agrobacterium tumefaciens, and induction of defense responses in plantlets via intact bacterial cells
Source: BMC Plant Biol. 2024 Feb 10;24:104. doi: 10.1186/s12870-024-04779-1 (PMC11297725; doi:10.1186/s12870-024-04779-1)
Supplement: Supplementary file 1 — Additional file 1: Supplementary Table 1. Analysis of variance (ANOVA) of relative defense enzymes activity in leaves of grapevine plantlets inoculated with endophytic bacteria and A. tumefaciens Gh1 after 0, 24, 48, and 72 h. Supplementary Table 2. Relative defense enzymes activity in leaves of grapevine after endophytic bacteria and A. tumefaciens Gh1 inoculation. [file 12870_2024_4779_MOESM1_ESM.docx]

**Supplementary Table 1** Analysis of variance (ANOVA) of relative defense enzymes activity in leaves of grapevine plantlets inoculated with endophytic bacteria and *A. tumefaciens* Gh1 after 0, 24, 48, and 72 hours.

| Source of variation |  | SOD | | | | PAL | | | | PPO | | | | POD | | | | TPC | | | |
| --- | --- | --- | --- | --- | --- | --- | --- | --- | --- | --- | --- | --- | --- | --- | --- | --- | --- | --- | --- | --- | --- |
|  | df | 0 h | 24 h | 48h | 72h | 0 h | 24 h | 48h | 72h | 0 h | 24 h | 48h | 72h | 0 h | 24 h | 48h | 72h | 0 h | 24 h | 48h | 72h |
| Treatment | 7 | 0.01 | 0.18** | 0.17** | 0.13** | 2.03 | 11503.7** | 12136.6** | 7507.4** | 0.007 | 0.96** | 0.89** | 0.54** | 0.335 | 19.5** | 147.18** | 69.90** | 0.088 | 4.83** | 19.75** | 5.53* |
| Erorr |  | 0.01 | 0.03 | 0.02 | 0.01 | 3.54 | 69.61 | 47.86 | 34.12 | 0.008 | 0.05 | 0.06 | 0.03 | 0.836 | 1.36 | 3.63 | 0.907 | 0.11 | 0.45 | 1.70 | 1.58 |
| Cv (%) |  | 11.95 | 12.77 | 9.72 | 8.53 | 8.25 | 8.95 | 7.31 | 7.49 | 13.27 | 12.70 | 13.98 | 10.30 | 10.23 | 10.16 | 12.5 | 7.3 | 5.377 | 8.814 | 14.64 | 15.38 |
| F-value |  | 0.86 | 6.48 | 9.15 | 10.55 | 0.57 | 166.40 | 253.56 | 220.05 | 0.89 | 19.70 | 16.12 | 19.98 | 0.40 | 14.35 | 40.47 | 77.02 | 0.76 | 10.74 | 11.57 | 3.50 |

*,** Significant at 1% and 5% probability level, respectively; df= Degrees of Freedom; Cv= Coefficient of variation

**Supplementary Table 2** Relative defense enzymes activity in leaves of grapevine after endophytic bacteria and *A. tumefaciens* Gh1 inoculation.

|  |  | Treatment | | | | | | | |
| --- | --- | --- | --- | --- | --- | --- | --- | --- | --- |
| Enzyme | Time (h) | Ctrl- | Ba35 | Ba47 | Ou55 | Ba35/Gh1 | Ba47/Gh1 | Ou55/Gh1 | Ctrl+ |
| TPC | 0 | 6.41+0.30 | 6.33+0.18 | 6.12+0.07 | 6.67+0.33 | 6.35+0.17 | 6.33+0.33 | 6.12+0.01 | 6.35+0.33 |
|  | 24 | 6.67+0.67 | 6.97+0.96 | 6.12+0.06 | 6.21+0.15 | 8.33+0.34 | 9.37+0.32 | 8.93+0.07 | 8.26+0.63 |
|  | 48 | 6.5+0.29 | 6.53+0.24 | 6.5+0.23 | 6.78+0.22 | 10.67+0.33 | 12.33+0.33 | 12.0+0.58 | 10.33+0.33 |
|  | 72 | 6.45+0.29 | 6.55+0.45 | 6.60+0.20 | 6.82+0.18 | 10.0+0.88 | 10.0+0.58 | 9.15+0.09 | 9.0 |
| PAL | 0 | 22.0+1.0 | 22.67+2.19 | 23.33+0.88 | 23.67+0.88 | 21.83+0.33 | 24.0+1.0 | 23.0+1.0 | 22+0.0 |
|  | 24 | 22.0+0.0 | 37.0+6.51 | 41.0+4.16 | 42.0+5.51 | 150.33+5.5 | 157+5.69 | 155.67+5.7 | 140.29+1.2 |
|  | 48 | 22.67+0.88 | 36.0+3.5 | 42.0+4.16 | 41.0+3.50 | 154.0+3.51 | 159.33+5.3 | 154.13+5.5 | 147.58+3.7 |
|  | 72 | 22.67+0.33 | 33.85+2.89 | 33.5+1.97 | 35.33+1.76 | 124.15+3.3 | 128.86+6.5 | 124.0+3.21 | 121.33+3.5 |
| POD | 0 | 8.47+0.27 | 9.08+0.51 | 9.17+0.44 | 8.83+0.60 | 9.0+0.58 | 8.67+0.28 | 8.74+0.63 | 9.54+0.72 |
|  | 24 | 8.20+0.06 | 9.77+0.15 | 9.20+0.15 | 9.63+0.33 | 14.50+0.29 | 14.60+1.73 | 13.15+0.60 | 12.76+0.14 |
|  | 48 | 8.30+0.06 | 9.41+0.29 | 9.72+0.15 | 9.34+0.24 | 22.66+1.77 | 23.0+0.58 | 24.22+2.44 | 15.33+0.33 |
|  | 72 | 8.30+0.03 | 8.67+0.36 | 8.89+0.49 | 8.73+0.15 | 17.82+0.10 | 19.19+0.81 | 18.0+0.58 | 13.48+1.01 |
| PPO | 0 | 0.57+0.017 | 0.68+0.03 | 0.66+0.01 | 0.64+0.02 | 0.67+0.05 | 0.73+0.08 | 0.72+0.09 | 0.67+0.04 |
|  | 24 | 0.60+0.01 | 1.65+0.052 | 1.42+0.05 | 1.56+0.06 | 2.33+0.12 | 1.92+0.13 | 2.27+0.15 | 2.14+0.26 |
|  | 48 | 0.60+0.02 | 1.49+0.16 | 1.49+0.20 | 1.41+0.20 | 2.17+0.11 | 2.16+0.1 | 2.17+0.04 | 1.94+0.15 |
|  | 72 | 0.66+0.10 | 1.44+0.06 | 1.57+0.04 | 1.53+0.06 | 1.89+0.13 | 1.99+0.11 | 1.85+0.11 | 1.81+0.11 |
| SOD | 0 | 0.81+0.01 | 0.92 + 0.04 | 0.95+0.02 | 0.85+0.10 | 0.88 + 0.10 | 0.99+0.07 | 0.93+ 0.04 | 0.93+0.06 |
|  | 24 | 0.90+0.01 | 1.23 +0.09 | 1.06 +0.04 | 1.18 +0.09 | 1.58+0.09 | 1.53+ 0.16 | 1.50 ***+***0.13 | 1.34 + 0.07 |
|  | 48 | 0.91+0.02 | 1.43 + 0.06 | 1.26 +0.04 | 1.38 +0.14 | 1.65 + 0.11 | 1.54 + 0.06 | 1.64 + 0.10 | 1.47 + 0.04 |
|  | 72 | 0.90+0.02 | 1.38 + 0.04 | 1.13+0.05 | 1.29+ 0.13 | 1.50 +0.07 | 1.46 +0.04 | 1.52 + 0.06 | 1.37 + 0.03 |
